# Investigating the Use of Virtual Reality Headsets for Postural Control Assessment: Instrument Validation Study

**DOI:** 10.2196/24950

**Published:** 2021-11-15

**Authors:** Brian Sylcott, Chia-Cheng Lin, Keith Williams, Mark Hinderaker

**Affiliations:** 1 Department of Engineering East Carolina University Greenville, NC United States; 2 Department of Physical Therapy East Carolina University Greenville, NC United States

**Keywords:** postural sway, virtual reality, force plate, center of pressure

## Abstract

**Background:**

Accurately measuring postural sway is an important part of balance assessment and rehabilitation. Although force plates give accurate measurements, their costs and space requirements make their use impractical in many situations.

**Objective:**

The work presented in this paper aimed to address this issue by validating a virtual reality (VR) headset as a relatively low-cost alternative to force plates for postural sway measurement. The HTC Vive (HTC Corporation) VR headset has built-in sensors that allow for position and orientation tracking, making it a potentially eﬀective tool for balance assessments.

**Methods:**

Participants in this study were asked to stand upright on a force plate (NeuroCom; Natus Medical Incorporated) while wearing the HTC Vive. Position data were collected from the headset and force plate simultaneously as participants experienced a custom-built VR environment that covered their entire field of view. The intraclass correlation coefficient (ICC) was used to examine the test-retest reliability of the postural control variables, which included the normalized path length, root mean square (RMS), and peak-to-peak (P2P) value. These were computed from the VR position output data and the center of pressure (COP) data from the force plate. Linear regression was used to investigate the correlations between the VR and force plate measurements.

**Results:**

Our results showed that the test-retest reliability of the RMS and P2P value of VR headset outputs (ICC: range 0.285-0.636) was similar to that of the RMS and P2P value of COP outputs (ICC: range 0.228-0.759). The linear regression between VR and COP measures showed significant correlations in RMSs and P2P values.

**Conclusions:**

Based on our results, the VR headset has the potential to be used for postural control measurements. However, the further development of software and testing protocols for balance assessments is needed.

## Introduction

An individual’s ability to maintain their balance is key for performing daily activities. One way to gauge an individual’s balance is to measure how much their center of pressure (COP) varies in the anteroposterior and mediolateral planes during quiet standing. This movement, which is referred to as *sway*, can be indirectly measured by using a force plate to record changes in the COP during quiet standing [1]. Postural sway in particular is an important indicator of an individual’s overall balance stability. As such, numerous studies have investigated the relationship between postural sway and physical activity performance [2]. However, the high costs and space requirements associated with the precision force plates used to measure postural sway can limit access to the use of the equipment for researchers and clinicians. There exists a need for a less resource-intensive solution.

A virtual reality (VR) system, which is defined as an interactive system that includes computers and media peripherals, can be used to create an environment that is similar to the real world and provide audio and video stimuli to users [3]. VR equipment used to be very expensive and used to require a lot of space. However, thanks to technological advances and flourishing innovations in the gaming industry, an affordable home-based VR headset with a built-in gyroscope and triaxis accelerometer for tracking the positions of users was developed to promote video games [4]. Research has shown that a home-based VR system can provide accurate data on motion and position changes (ie, for clinical and research purposes) that are as accurate as those provided by a significantly more expensive kinematic motion capture system [5]. Further, although Niehorster et al [6] did not suggest the use of a home-based VR system for scientific experiments due to the system’s lower latency at the millisecond level, Niehorster et al [6] did report that the precision of the VR tracking measurements was high. For balance measurements in clinical and lab settings, highly precise position data is more important than low latency. A study also proposed using a VR system to improve balance function [7]. Additionally, home-based VR systems have been proven to have reasonably accurate position tracking [6] and spinal mobility measurement [8] functions.

In a lab or clinic setting, expensive force plates are used to quantify balance performance. However, their costs and space requirements prevent the use of a force platform in home settings for health care purposes. A home-based VR system has the advantage of providing balance measurements via the VR headset’s outputs [9]. Studies have investigated using VR headsets to assess human balance performance based on the inverted pendulum model of balance [10]. These studies’ results validated the use of VR headset outputs as a method for quantifying postural sway in balance performance assessments [9]. However, the test-retest reliability of this method has not been reported, and it is unknown if the same results can be generated by a different brand of VR headset.

The goal of this study was to investigate the use of a VR headset (HTC Vive; HTC Corporation) as a cost-effective alternative to a force plate for measuring postural sway. If a VR headset’s motion tracking equipment was valid for use in measuring values such as postural sway variables, many options for researching the effects that VR environments have on an individual’s postural sway and balance without the need for extra equipment would become available. VR headsets could eventually become clinical tools that clinicians can use for balance assessments in a clinic or home setting. In this study, we compared the position outputs from a VR headset to the COP readings from a force plate to determine if the headset can effectively measure balance control.

## Methods

### Participants

A total of 20 healthy participants (age: mean 45 years, SD 26 years) were asked to participate in this study after institutional review board approval was received. Physical health screening was performed on all participants to exclude anyone with balance issues, dizziness, or mobility deﬁcits. After receiving informed consent from each participant, screening was conducted to exclude individuals with neurological and orthopedic disorders and any potential balance or dizziness issues.

### Procedure

**Figure 1:**
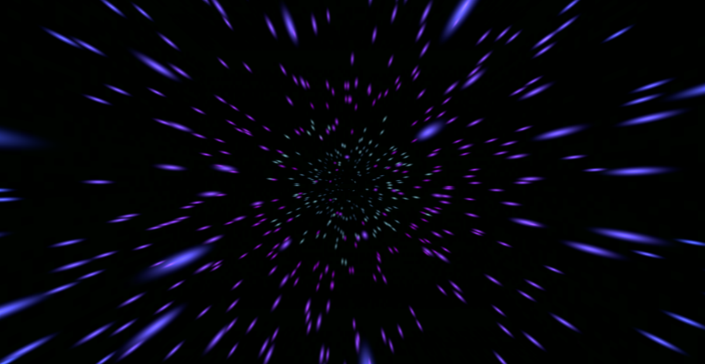
A virtual scene that was displayed during the trials for the eyes open condition.

The participants were asked to stand quietly, either with their eyes open or with their eyes closed, on a force plate (NeuroCom; Natus Medical Incorporated) while wearing the HTC Vive. Three 20-second trials were performed for the eyes open and eyes closed conditions. During the trials for the eyes open condition, a static virtual scene was shown to the participants to reduce visual feedback and to help them maintain their balance. Figure 1 shows the virtual scene (Unity version 2018.3.8; Unity Technologies) that was seen by the participants of this study.

The COP data were recorded by the force plate at a sampling frequency of 200 Hz. The VR position data were recorded at a sampling frequency of 10 Hz. The COP and VR position data were computed to find the normalized path length (NPL) [11], root mean square (RMS) [11], and peak-to-peak (P2P) value [11] for sway in the medial-lateral and anterior-posterior directions by using a customized MATLAB code (MathWorks). The NPL, RMS, and P2P value were calculated as follows:




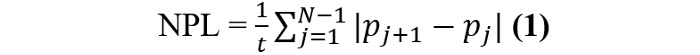







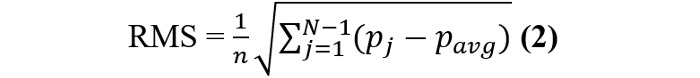







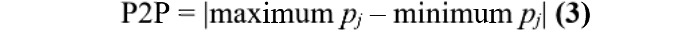




In these equations, *t* is the time duration, *N* is the number of samples, *p_avg_* is the mean of the data, and *p_j_* is either COP data or VR data at time sample *j*.

The test-retest reliability of the NPLs, RMSs, and P2P values in the three trials for each test condition were examined by using intraclass correlation coefficients (ICCs) and 95% CIs. A 2-way mixed-effects analysis of variance was conducted with a model comprised of the subject and trial numbers. ICCs were classified as poor (<0.5), moderate (0.5-0.75), good (0.75-0.9), or excellent (>0.90) [12].

The strength of the association between the COP and VR outputs was computed via linear regression by using the NPL, RMS, and P2P value of the COP data as the dependent variables and the VR output as the univariate predictor for each measure separately. The data from the first trial and the 3-trial averages were analyzed. Coefficients of determination (*R*^2^) were reported as the amount of variance in COP data, which was estimated by using the VR outputs. The significance level was set to α<.05. IBM SPSS Statistics software (version 25.0.0.1; IBM Corporation) was used to conduct the statistical analysis.

## Results

**Figure 2:**
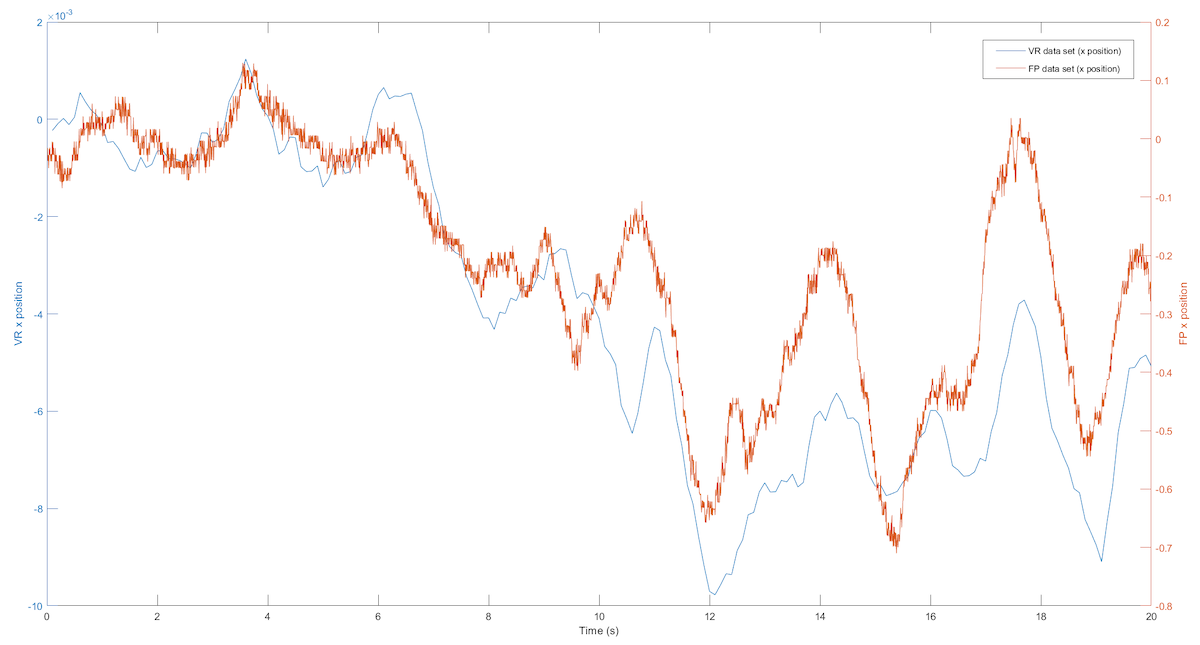
The blue line represents the VR data, and the green line represents the center of pressure data from the FP for sway in the medial-lateral direction. FP: force plate; VR: virtual reality.

**Figure 3:**
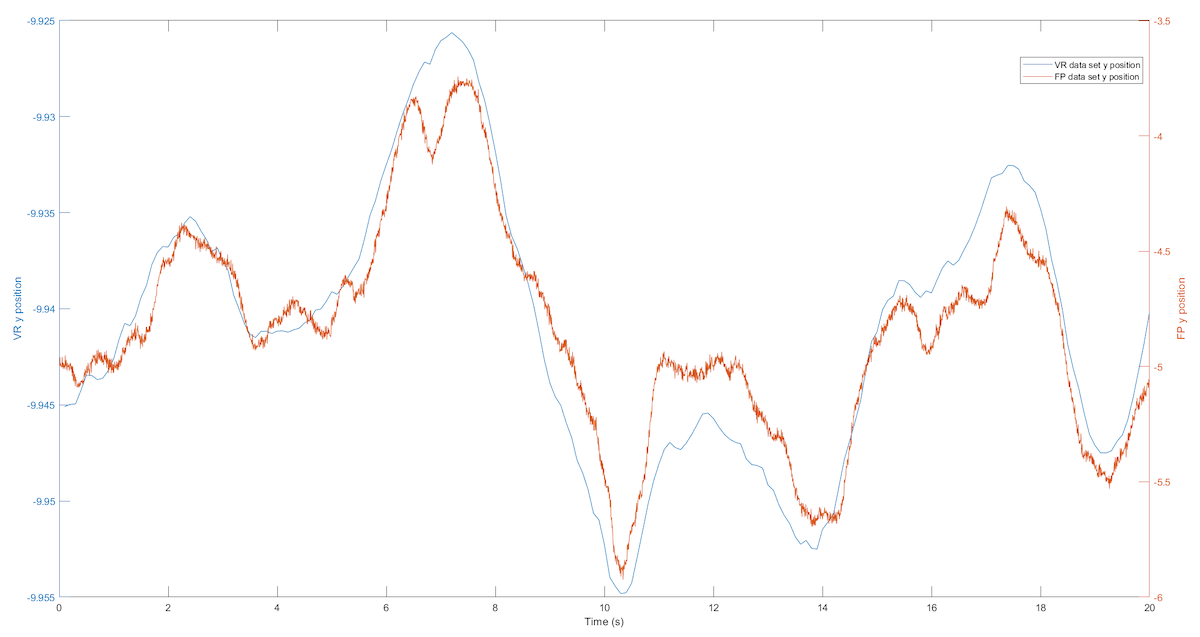
The blue line represents the VR data, and the green line represents the center of pressure data from the force plate for sway in the anterior-posterior direction. FP: force plate; VR: virtual reality.

We first examined the COP and VR position trajectory. Examples of the raw medial-lateral and anterior-posterior displacement data collected from the force plate and the VR headset are shown in Figure 2 and Figure 3. The COP and VR outputs showed similar patterns for sway in the medial-lateral and anterior-posterior directions during a test trial.

**Figure 4:**
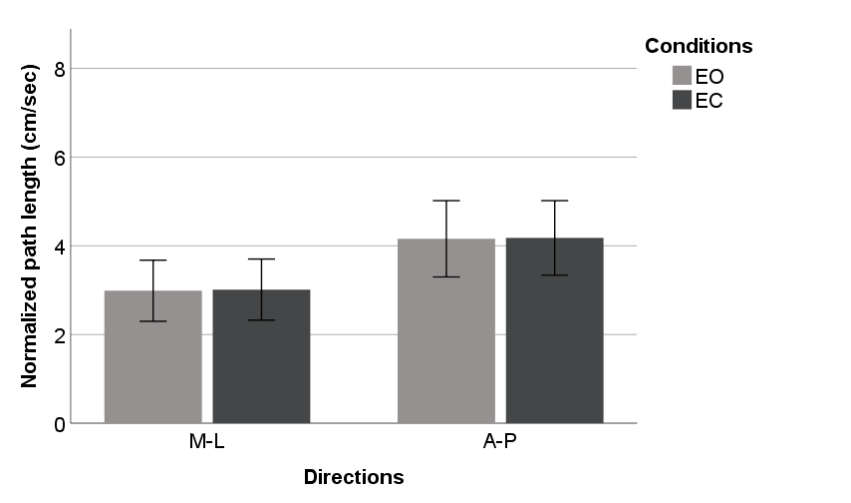
Center of pressure outputs (means and 1 SD) by postural sway direction for normalized path length. A-P: anterior-posterior; EC: eyes closed; EO: eyes open; M-L: medial-lateral.

**Figure 5:**
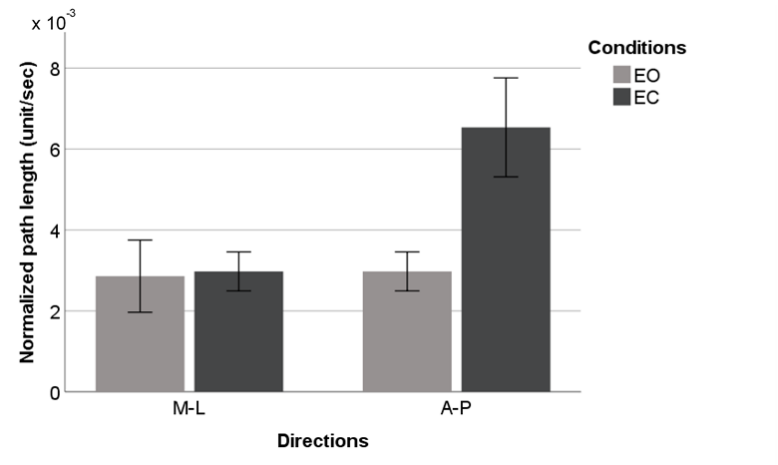
Virtual reality headset outputs (means and 1 SD) by postural sway direction for normalized path length. A-P: anterior-posterior; EC: eyes closed; EO: eyes open; M-L: medial-lateral.

**Figure 6:**
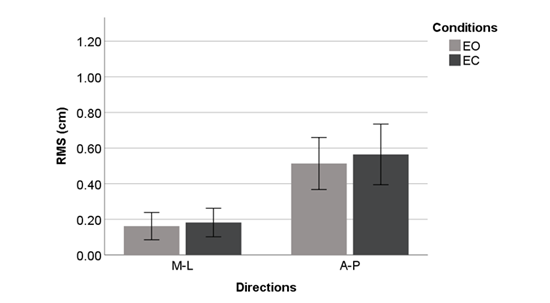
Center of pressure outputs (means and 1 SD) by postural sway direction for the center of pressure RMS. A-P: anterior-posterior; EC: eyes closed; EO: eyes open; M-L: medial-lateral; RMS: root mean square.

**Figure 7:**
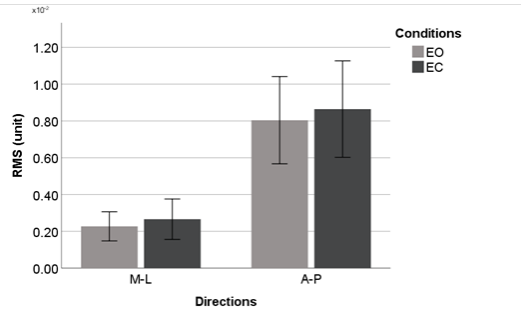
Virtual reality headset outputs (means and 1 SD) by postural sway direction for the virtual reality RMS. A-P: anterior-posterior; EC: eyes closed; EO: eyes open; M-L: medial-lateral; RMS: root mean square.

**Figure 8:**
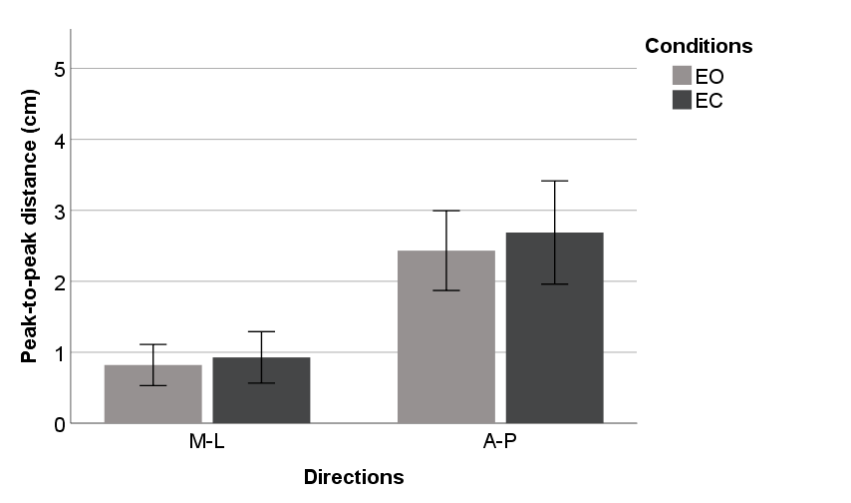
Center of pressure outputs (means and 1 SD) by postural sway direction for the peak-to-peak value. A-P: anterior-posterior; EC: eyes closed; EO: eyes open; M-L: medial-lateral.

**Figure 9:**
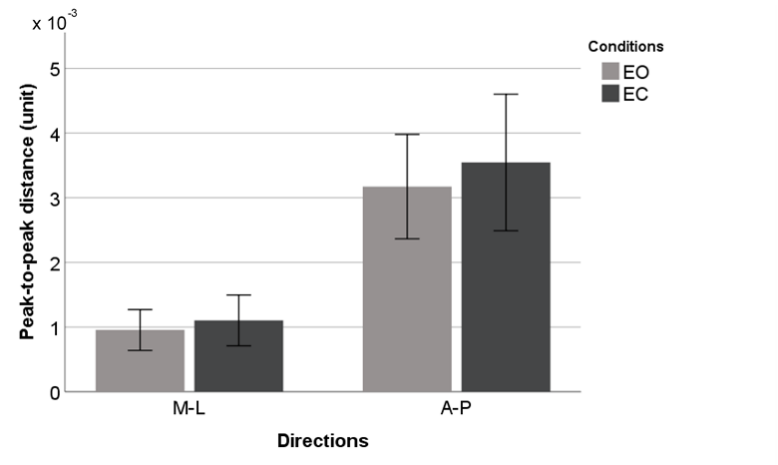
Virtual reality headset outputs (means and 1 SD) by postural sway direction for peak-to-peak value. A-P: anterior-posterior; EC: eyes closed; EO: eyes open; M-L: medial-lateral.

Figures 4-9 summarize the results of the COP and VR measurements for the two postural sway directions, which were taken during the trials for the eyes open and eyes closed conditions for COP and VR measurements; the bars represent the 3-trial averages for sway in the medial-lateral and anterior-posterior directions and the eyes open and eyes closed conditions. The test-retest reliability of the RMS and *P* value of VR outputs was as good as that of the RMS and P2P value of force plate outputs. However, the test-retest reliability of the NPL of VR outputs was slightly worse than that of the NPL of force plate outputs (Table 1). Among the COP outputs, the NPL had the highest test-retest reliability (ICC: range 0.982-0.997), followed by the RMS (ICC: range 0.360-0.740) and P2P value (ICC: range 0.228-0.759). The test-retest reliability of the NPL, RMS, and P2P value of VR outputs, which ranged from 0.448 to 0.763, 0.285 to 0.574, and 0.348 to 0.636, respectively, was similar. VR data were significantly associated with the COP data for the RMS and P2P value (Table 2). In the first trial, the RMS and P2P value of VR outputs had a strong association with the RMS and P2P value of COP data. The 3-trial averages for the same VR output measures also had a strong association with the corresponding 3-trial averages for COP data. However, medial-lateral postural sway and the standing with eyes open condition were not strongly associated with the RMS and P2P value of COP data. The coefficients of determination for the NPL of VR outputs were lower than those for the RMS and P2P value of VR outputs. Swaying in the anterior-posterior direction and standing with eyes closed had a strong association with the COP data for the RMS and P2P value.

**Table 1:** The test-retest reliability in the three trials. The intraclass correlation coefficients and 95% CIs were calculated for the normalized path length (NPL), root mean square (RMS), and peak-to-peak (P2P) value of the force plate (FP) and virtual reality (VR) outputs for sway in the medial-lateral (M-L) and anterior-posterior (A-P) directions and the eyes open (EO) and eyes closed (EC) conditions.

Condition (sway direction)	Intraclass correlation coefficients (95% CI)					
	NPL of FP outputs	NPL of VR outputs	RMS of FP outputs	RMS of VR outputs	P2P value of FP outputs	P2P value of VR outputs
EO (M-L)	0.994 (0.987-0.997)	0.448 (0-0.771)	0.360 (0-0.726)	0.496 (0-0.786)	0.228 (0-0.673)	0.579 (0.102-0.824)
EC (M-L)	0.997 (0.994-0.999)	0.689 (0.341-0.870)	0.740 (0.462-0.888)	0.574 (0.124-0.819)	0.759 (0.503-0.896)	0.636 (0.241-0.846)
EO (A-P)	0.989 (0.977-0.995)	0.576 (0.100-0.823)	0.523 (0-0.802)	0.520 (0-0.802)	0.469 (0-0.775)	0.481 (0-0.777)
EC (A-P)	0.992 (0.984-0.997)	0.763 (0.492-0.902)	0.499 (0-0.787)	0.285 (0-0.704)	0.428 (0-0.758)	0.348 (0-0.732)

**Table 2:** Linear regression coefficients of determination for the center of pressure (COP) values predicted by the virtual reality (VR) headset outputs for each condition (COP and VR outcomes of the first trial and the 3-trial average of outcomes). Coefficients for the normalized path length (NPL), root mean square (RMS), and peak-to-peak (P2P) value of the force plate and VR outputs for sway in the medial-lateral (M-L) and anterior-posterior (A-P) directions and the eyes open (EO) and eyes closed (EC) conditions are shown.

COP and VR outcomes		Coefficients of determination, *R^2^*			
		EO condition and M-L sway	EO condition and A-P sway	EC condition and M-L sway	EC condition and A-P sway
**NPL**					
	First trial	0.005	0.024	0.004	0.001
	3-trial average	0.089	0.038	0.013	0.038
**RMS**					
	First trial	0.173	0.891^a^	0.449^b^	0.887^a^
	3-trial average	0.735^a^	0.862^a^	0.556^a^	0.934^a^
**P2P**					
	First trial	0.626^a^	0.734^b^	0.418^b^	0.840^a^
	3-trial average	0.718^a^	0.769^b^	0.608^a^	0.890^a^

^a^Significant the *P*<.001 level.

^b^Significant at the *P*<.05 level.

## Discussion

### Principal Findings

The test-retest reliability of VR position outputs was similar to that of COP data for the RMS and P2P value. Our results suggest that the position outputs from the VR headset had a strong correlation with postural sway variables, such as the RMS and P2P value computed from the COP data. However, the NPL of VR outputs had a weak correlation with the NPL of COP outputs, and this might have been due to the characteristics of VR position data and COP measurements. Force plates take COP measurements from below an individual’s center of gravity, and VR headsets take position measurements from above an individual’s center of gravity. Therefore, postural sway in an individual would have had a greater impact on the headset’s measurements than on the force plate’s measurements, resulting in weaker correlations in NPLs.

A different brand of VR headset was validated for measuring balance, and the results showed good to excellent correlations between COP and VR headset outputs [9]. Our study demonstrated similar results for a different brand of VR headset. Moreover, our data further validated the test-retest reliability of the VR headset we used. The Wii Balance Board (Nintendo) was used to take COP measurements in a study by Marchetto and Wright [9]. The Wii Balance Board was designed as a video game controller with a low sampling frequency (40 Hz) [13]. In our study, a laboratory-grade force platform was used to collect the COP data. Although the Wii Balance Board was validated for taking COP measurements, a laboratory-grade force platform may provide more accurate data, especially in studies that validate other devices for balance assessments [13,14].

The average ICC was better in the trials for the eyes closed condition. This may have been due to the lack of variability in postural sway when standing with eyes open [15]. The ICC values may improve if variables with larger variations or more challenging balance variables, such as optic flow, are analyzed. Moreover, the sample size is another key factor that affects the ICC. Having a larger sample size may help to improve the test-retest reliability of VR position outputs.

This study presents promising results that indicate the usefulness of a VR headset as an alternative device for measuring balance control. Overall, the position data that were recorded by the VR headset correlated strongly with the COP data that were recorded by the force plate. The RMSs and P2P values of the data seem to indicate that there may be magnitude differences between the position data recorded by the headset and the COP data recorded by the force plate. Future work could be conducted to establish how much of a magnitude difference this is and if further data manipulation is necessary to obtain better correlations between the COP and position data.

Although the data show promise, there is still a need for improvement. In several trials, the correlation values were lower than 0.2. This indicated weak correlations among data sets. Since these correlation values tended to be outliers, it is likely that these lower values stemmed from calibration or procedure issues. The data analyzed in these trials also may have exhibited little variability, and this could have contributed to the low correlation values. Further software development for taking measurements by using the headset can be conducted, and improvements to the software used to analyze the data can be made. The further calibration of the headset and other data collection devices may also improve results. Adjustments can be made to the procedures used during data collection, such as syncing the headset and force plate to start data collection at the same time by inputting the same input command on a single PC. Future work can be conducted to investigate the correlations between position data recorded by VR headsets and position data recorded by motion capture systems. Comparisons between such data can be conducted to further support the use of a VR headset as a clinical and research tool.

### Conclusion

Using VR position outputs could be an alternative way of measuring postural sway. However, a standard method will need to be established before such data can be used in this manner. Overall, VR headset position outputs appear to have good potential for being used in balance control studies. The lower cost of a VR headset system is an advantage and promotes the use of this device in clinic settings. However, further validation and software development may be needed.

### Key Points

An affordable home-based VR headset with a built-in gyroscope and triaxis accelerometer for tracking the position of a user was developed to promote video games. The goal of this study was to investigate the use of a VR headset as a cost-effective alternative to a force plate for measuring postural sway. The test-retest reliability of the VR headset and the postural control variables that were computed by the VR headset and a laboratory-grade force platform were compared. The lower cost of a VR headset system is an advantage and promotes the use of this device in measuring postural sway. However, further validation and software development may be needed.

## Abbreviations

COP: center of pressure
ICC: intraclass correlation coefficient
NPL: normalized path length
P2P: peak-to-peak
RMS: root mean square
VR: virtual reality
